# Clinical Manifestations, Diagnosis and Management of Synovial Fistula Associated Lateral Ankle Sprain or Instability: A Retrospective Study of 19 Surgically Confirmed Patients

**DOI:** 10.3390/ijerph19042428

**Published:** 2022-02-19

**Authors:** Jahyung Kim, Bum-Jin Shim, Jae-Shin Yang, Altanzul Bat-Ulzii, Jaeho Cho

**Affiliations:** 1 Department of Orthopaedic Surgery, Armed Force Gangneung Hospital, Gangneung 25422, Korea; hpsyndrome@naver.com; 2 Department of Orthopaedic Surgery, Chuncheon Sacred Heart Hospital, Hallym University, Chuncheon 24253, Korea; redpross@naver.com (B.-J.S.); yummysilver@naver.com (J.-S.Y.); 3 Institute for Skeletal Aging and Orthopedic Surgery, Chuncheon Sacred Heart Hospital, Hallym University, Chuncheon 24253, Korea; azulaazulaa@gmail.com

**Keywords:** ankle, synovial fistula, ankle sprain, lateral ankle instability

## Abstract

We aimed to investigate the preoperative history, clinical manifestations, imaging findings, and postoperative clinical outcomes for patients with surgically confirmed synovial fistula around the ankle joint. 19 consecutive patients who were confirmed to have synovial fistula in the surgical field were enrolled in this study. Medical records of all patients in terms of preoperative details, operative findings, and postoperative outcomes at 1 year after the surgery were retrieved. As a diagnostic modality, the normal saline test or MRI was used. Intraoperatively, the synovial fistula was repaired with the capsuloligamentous repair or additional periosteal augmentation. All patients had a history of ankle sprain prior to symptoms and showed positive results in the saline load test. One patient had recurred symptom after the surgery, so needed a revisional periosteal augmentation. At 1 year follow-up period, the average Foot ankle outcome score was 87.65 and no surgery-related complication was detected. Synovial fistula of the ankle joint needs to be taken into consideration as a possible complication in patients with ankle sprain history and recurrent joint swelling. The saline load test would be useful for its diagnosis, and treatment should be focused on the complete closure of capsular opening along with restoration of its surrounding pathologic conditions.

## 1. Introduction

Synovial fistula (SF) of joint is a rare pathologic condition that is formed through a defect within the joint capsule. There have been several reports of SF around the knee, shoulder, and wrist joints, which developed in the wake of intra-articular surgeries such as arthroscopic procedures [1,2,3,4,5,6,7]. Regarding its mechanism, SF operates as a check-valve and drains the synovial fluid externally into the extracapsular space. If an excessive amount of synovial fluid is drained externally, SF can also be communicated to an adjacent bursal sac [8,9,10]. In other words, the SF is generally presumed to be iatrogenic and can accompany a communication with adjacent extracapsular spaces including bursal sac. Although not many cases of SF of the ankle joint have been reported, most of these were found in association with adjacent bursitis [11,12,13].

The lateral ankle sprain is known to be the most common form of ankle injury, with an estimated more than 23,000 ankle sprains occurring daily in the United States, which is equal to one sprain per 10,000 persons daily [14]. Although most of lateral ankle sprains heal without problems over time, residual symptoms such as persistent pain or instability occur approximately 15% to 20% of these patients [15,16]. Furthermore, the mechanical instability after lateral ankle sprain may lead to anatomic changes including pathologic laxity of joint, synovial changes, and development of degenerative joint condition [17]. Since SF of the joint can occur through the defects made on the altered joint capsule condition, we hypothesized that SF in the ankle joint may also be formed as a result of lateral ankle sprain [18].

To the best of our knowledge, no published clinical studies have reported the clinical manifestations or features, diagnostic tools, imaging findings, and surgical procedures and outcomes for SF associated lateral ankle sprains or instability. The primary aim of the present study was to investigate the preoperative history, clinical manifestations, or imaging findings for 19 patients with surgically confirmed SF around the ankle joint. Furthermore, clinical outcomes following surgical procedures were also evaluated. 

## 2. Materials and Methods

This retrospective study was approved by the medical ethics committee at our institution (Institutional Review Board number: CHUNCHEON 2021-07-010) and written informed consents for publication of this report were obtained from all included patients. 

Nineteen consecutive patients with recurrent ankle swelling around the lateral ankle, who were confirmed to have SF in the surgical field between 2016 January and 2020 December were enrolled in this study. The swelling was aggravated during motion and relieved at rest, without a history of repetitive irritation over lateral malleolus and noticeable signs indicating infection. Medical records of all patients were reviewed, and the following details were retrieved: age, gender, trauma history, time to visit after trauma, imaging finding, performed surgical procedures, and complications after surgery. 

**Figure 1:**
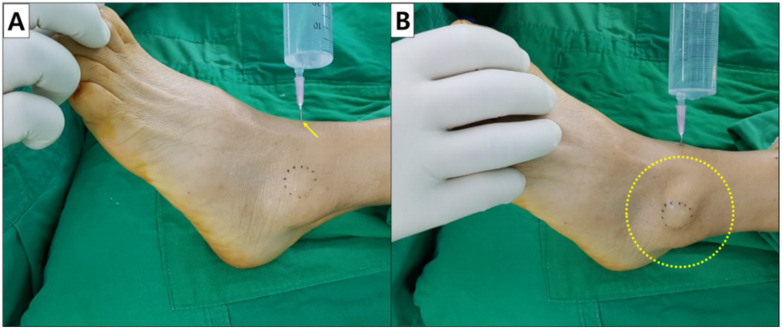
Saline load test. (**A**) An 18-gauge needle is inserted at the ankle joint where a typical anterior medial portal for ankle arthroscopy is created (Asterick). (**B**) Following a normal saline injection, the ankle joint is manipulated through its range of motion. The test is considered positive if soft tissue around the lateral malleolar area (Dotted circle) becomes dilated.

In all patients, the saline load test, a common tool used to evaluate traumatic arthrotomies of the knee, was modified and applied on the ankle joint [19]. A puncture was made using an 18-gauge needle at the ankle joint where a typical anterior medial portal for an ankle arthroscopy is created. The needle was inserted through a soft spot over the ankle joint line. After confirming the intra-articular placement of the needle tip, 30 mL of sterile normal saline solution was injected into the ankle joint. Then, the ankle was manipulated through its range of motion and a soft tissue dilation around the lateral malleolar area was observed for extravasation of fluid. If this occurred, the test was defined to be positive (Figure 1).

If patients agreed to receive additional imaging studies, MRIs were taken to localize the herniated joint capsule, evaluate surrounding ligament status, and detect concurrent bursitis. Patients were followed up for at least one year after the surgery, and clinical outcomes at the last follow up period were evaluated according to Foot and ankle outcome score (FAOS) [20]. Postoperative complications including a recurrence of SF were also checked. A recurrence of SF was suspected when the patient complained again with preoperative symptoms and the saline load test confirmed the SF. 

### 2.1. Surgical Technique 

**Figure 2:**
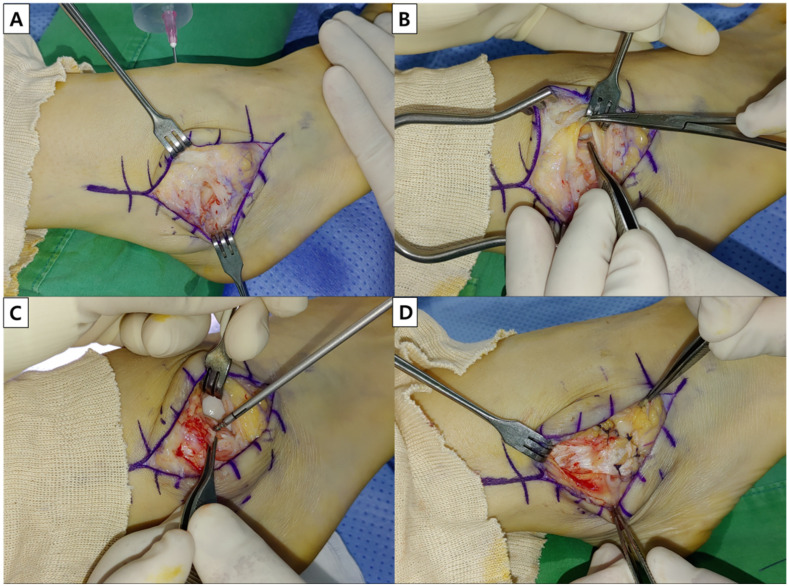
Capsuloligamentous repair. (**A**) The saline load test is performed to detect the opening of the synovial fistula. (**B**) The capsular opening of the synovial fistula is identified. (**C**) In case the opening is located close to the fibula, the anchor is approached on the anteroinferior aspect of fibula to facilitate the closure. (**D**) Meticulous closure of the capsular opening is performed.

**Figure 3:**
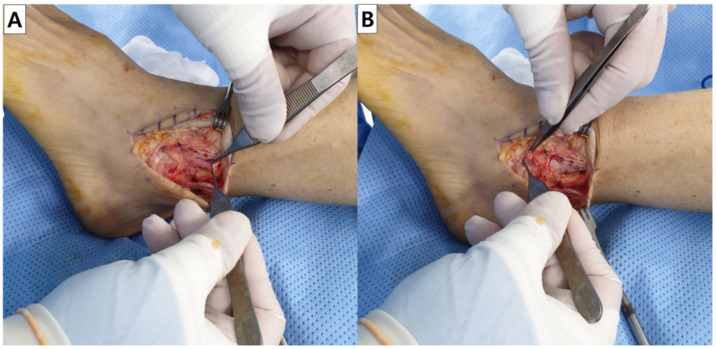
Periosteal augmentation. (**A**) The rectangular periosteal flap of a size sufficient to cover the capsular opening of synovial fistula was dissected and isolated from the fibula. (**B**) The flap is turned down proximally to distally and reinforced over the capsular defect using sutures or anchors.

Surgical treatment was performed by a single surgeon (J.C.). After anesthesia and sterile draping, a longitudinal midline incision was made on the lateral malleolus. Following close evaluation of the capsular and ligamentous status around the fibula, the saline load test was performed to detect the saline coming out of capsular opening of the SF. In case there was accompanying bursitis, a bursectomy was performed first, taking care of not to excessively detach the surrounding structures. Then, the capsular opening was closed using sutures or anchors, along with imbrication of ligaments and reattachment of the extensor retinaculum to the fibula, which is known as the modified Brostrom operation (Figure 2). In case the soft tissue around the SF was not sufficient, the opening was reinforced using a periosteal augmentation (Figure 3). At the end of the procedure, the saline load test was performed lastly to ensure complete closure of opening of the SF.

### 2.2. Postoperative Protocol 

The operated limb ankle was immobilized with a short leg cast for 4 weeks. During this period, tolerable weight bearing was allowed after 2 weeks of non-weight bearing. After four weeks, the cast was removed, and range of motion was initiated using a functional brace. From 8 weeks postoperatively, patients were allowed to return to daily activities.

## 3. Results 

**Figure 4:**
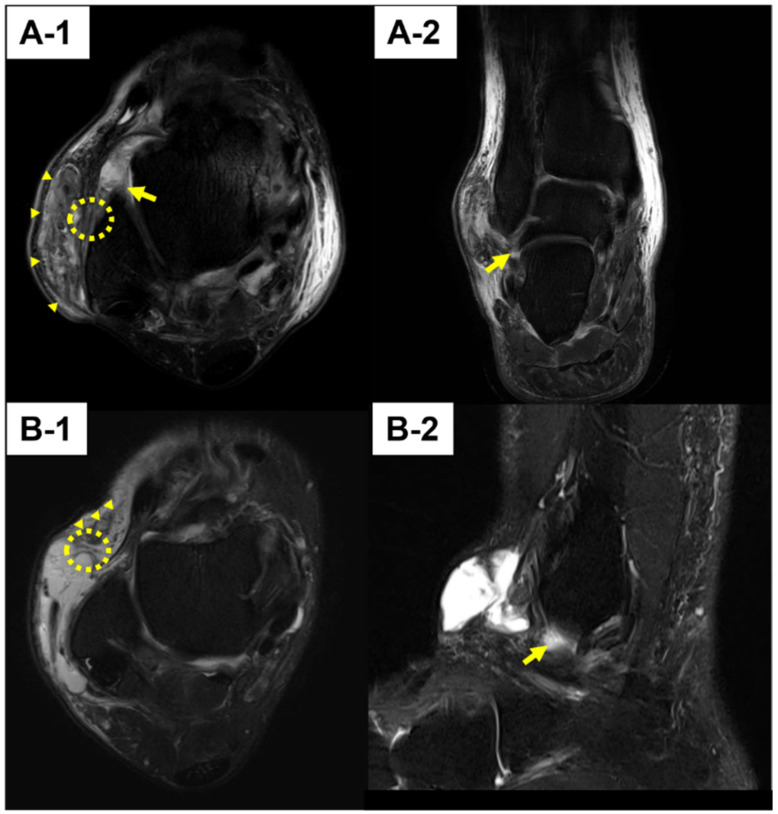
MRI findings. (**A-1**) Bursitis, located on the lateral malleolar area (Tailless arrows), connected with the synovial fistula (Dotted circle), along with chronic anterior talofibular ligament rupture (Arrow). (**A-2**) Along with lateral malleolar bursitis connected with synovial fistula, chronic rupture of calcaneofibular ligament (Arrow). (**B-1**) Bursitis located on the premalleolar area (Tailless arrows), connected with the synovial fistula (dotted circle). (**B-2**) Along with premalleolar bursitis connected with synovial fistula, chronic rupture of anterior talofibular ligament (Arrow).

Out of nineteen patients, eight were male and eleven were female. The mean age at diagnosis was 57.8 years (range, 32–82 years). All patients had a history of ankle sprain prior to symptoms. The average time to visit after sprain was 12.9 months (range, 3–84 months). The saline load test was positive in all patients. MRI was taken in 13 patients who agreed to receive an additional diagnostic study before surgery, and a variety of MRI findings including chronic ligament rupture, bursitis, and connection between SF and bursal sac could be detected (Figure 4). As for the ligament status, anterior talofibular ligament was torn in all included patients and calcaneofibular ligament was torn in 7 patients, which means that both ligaments were injured in 7 out of 13 patients.

Regarding operative findings, bursitis could be detected along with SF in 11 patients while it could not be found around SF in 8 patients. Out of 11 cases with bursitis, 8 were located on the premalleolar area and 3 were on the lateral malleolar area. In addition, 9 had a communication with SF, while the other two did not. Specifically, all the bursal sacs located on the premalleolar area were communicated with SF, while two out of three sacs on the lateral malleolar area existed without communication with SF. 

Twelve patients were operated with capsuloligamentous repair and additional periosteal augmentation was required in seven patients. In one patient repaired with capsuloligamentous repair, preoperatively complained symptom recurred at 3 months after the surgery. Accordingly, a revision surgery with periosteal augmentation was performed. All patients were followed up for one year and the average FAOS score at this period was 87.66 (range 77.4 to 98.15), without notable surgery-related complications (Table 1).

### Illustrative Case

**Figure 5:**
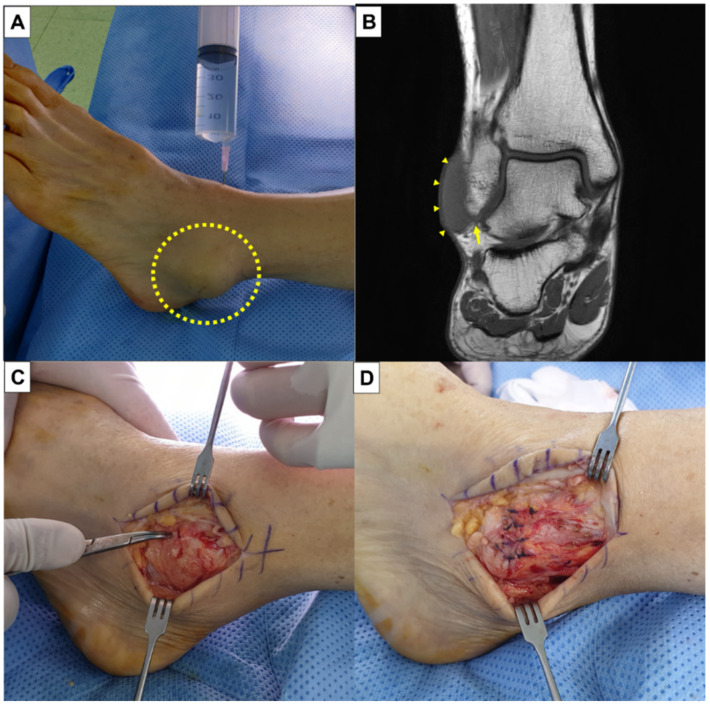
Illustrative case (**A**) A positive saline load test (dotted circle). (**B**) MRI finding showing a cystic lesion (tailless arrows) which is communicated with the joint around the fibular tip (arrow), without any evidence of bursitis. (**C**) A sizable synovial opening on the weakened portion of the joint capsule is detected (pointed with a mosquito forceps). (**D**) A capsuloligamentous repair along with periosteal augmentation is performed.

A 52-year-old female patient complained of recurrent swelling of the ankle joint that was noticed 6 months after ankle sprain. The symptom was on the premalleolar area, which was aggravated during motion and relieved at rest. There was no severe pain, tenderness, nor noticeable infection sign around the swollen area. Blood samples also revealed normal. There was an instability on the ankle joint and the saline load test turned out to be positive. The patient was evaluated with an MRI, in which the joint was communicated with the premalleolar lesion around the fibular tip, without any evidence of bursitis. Surgery was performed under spinal anesthesia. Following a skin incision, a cavity filled with serous liquid was found immediately under the dermal plane. After meticulous soft tissue dissection, a sizable synovial opening on the weakened portion of the joint capsule could be detected. A succeeding saline load test also showed fluid leakage through the opening. Using a 3.0 mm Bio Suture anchor (Arthrex Inc., Naples, FL, USA) the capsuloligamentous repair was performed. Because the repair was insufficient to completely cover the synovial opening due to excessive tissue defects, additional periosteal augmentation was needed (Figure 5). A confirmative saline load test revealed a complete closure of the capsular opening. At the 12 months follow-up, the patient showed satisfactory outcomes of average FAOS score of 94.88, without any evidence of recurrence.

## 4. Discussion

When a patient presents to a clinic with diffuse swelling over the lateral malleolar area, adventitious malleolar bursitis would probably be a diagnosis that is generally thought of by most orthopaedic surgeons. It is an accumulation of fluid within a bursa, which is formed by repetitive mechanical stimulations around the malleolar area [21]. However, patients sometimes demonstrate recurrent swelling anterior to the lateral malleolar area, so-called premalleolar area, that is aggravated with motion and relieved at rest, without a history of repetitive irritation over lateral malleolus or noticeable signs indicating infection [22]. Since the magnitude of swelling differs upon ankle motion, it can be estimated that the swollen area is closely connected to the ankle joint via a communicating tract, which is believed to be a SF. Since SF acts in accordance with joint motion and muscle contracture, recurrent and joint motion-dependent ankle swelling should be approached differently from adventitious bursitis [18]. 

The interesting fact is that upon detailed history taking, these patients recalled that the symptom occurred after suffering a serious traumatic event, specifically, an inversion-plantar flexion ankle injury. It is the most common type of ankle injury which results in strain to the lateral ankle structures [23]. If the strain exceeds the tensile strength of the tissues, anatomical changes on the ankle joint structures may occur, which can contribute to joint instability or synovial and degenerative changes [17]. As a result, it can be estimated that such altered ankle joint biomechanics may lead to defects on the weakened part of the joint capsule, and eventually create a SF. To the best of our knowledge, there is only one report in the Korean literature that proposed the possibility of a lateral ankle sprain-induced SF along with the surgical outcomes of seven patients [24]. However, it did not concretely describe the clinical features, diagnostic measures, imaging findings, and follow-up postoperative outcomes of SF. 

As far as we are concerned, only a few case reports regarding SF after ankle sprain have been reported in the English literature. Naito et al. reported a patient with lateral premalleolar bursitis of the ankle associated with the lateral ankle instability and suggested that the check-valve mechanism of the SF was exacerbated by the joint instability [25]. In this case, however, the SF was communicated with tendon sheath rather than the bursal cavity and a description whether the symptom differed upon joint motion was not given. In other words, it can be estimated that although the SF was developed as a sequel to ankle instability, the bursitis itself was not communicated with SF. Similarly, Tonogai et al. reported a case of lateral premalleolar bursitis caused by chronic ankle instability, without enough symptomatic, imaging, and operative field details indicating communication between the ankle joint and bursal cavity [26]. Besides, the patient had concurrent ankle osteoarthritis and was treated with endoscopic bursectomy and joint salvage procedure, instead of SF closure. Consequently, we believe that the present study is the first to consecutively describe various disease entities of SF after lateral ankle sprain.

In terms of disease entity, we could detect various types of the SF. Reviewing the patients confirmed to have SF intra-operatively, ten patients had visible bursa before the surgery while nine patients did not. Instead, these nine patients complained of recurrent joint swelling that occurs in the joint range of motion and disappears in rest. It could be estimated that the SF was developed after ankle sprain, yet the herniated joint fluid did not form a bursal sac. Additionally, three cases of bursitis were located right over the lateral malleolus. Although it is a common location of an adventitious malleolar bursitis, all three cases had SF and one bursal sac was communicated with the ankle joint. Accordingly, even if the bursitis does not exist or takes a form of adventitious malleolar bursitis, attention should be paid not to ignore SF if a patient has a history of an ankle sprain.

Once SF is suspected, we prefer the saline load test to confirm its presence. The saline load test, typically used to identify traumatic arthrotomy of joints, can be performed easily at the outpatient clinic to indirectly detect the presence of SF [19,27]. If the soft tissue around the area the patient experienced symptoms becomes dilated after a normal saline injection with simultaneous manipulation of the ankle joint through a range of motion, operative closure of the SF should be considered. As noted by Lee et al., this test can also be used to locate the capsular opening during surgery and to check for extravasation after SF closure. [13]. Additionally, imaging studies like ultrasonography, CT arthrogram, and MRI can be used along with the saline load test. These are helpful to estimate the size and location of the SF, evaluate surrounding ligament status, and detect concurrent bursal sac [13,25,28]. 

The surgical goal for SF should be mainly on restoration of trauma-derived pathologic conditions, to both eliminate symptoms and prevent recurrence. In other words, tight closure of the capsular defect and solid stabilization of the detached ligament must be confirmed. In this context, we believe that if a patient with unstable ankle has a concurrent SF, the modified Brostrom operation should be performed in an open manner rather than an arthroscopic one to effectively close the capsular opening. If soft tissue around the defect was sufficient, the capsular opening and torn ligament were repaired with absorbable sutures or anchors. In some cases, however, the remaining capsule was not enough to cover the capsular opening itself, either because of thinned joint capsule or insufficient remnant soft tissue after an extensive bursectomy. In these instances, a periosteal flap, turned down from proximally to distally, was used to augment the defect [29]. As a matter of fact, we experienced one patient who had recurred symptoms after capsule and ligament repair, probably because of insufficient coverage of capsular defect. A revision surgery using a periosteal augmentation was performed and no recurrence was reported since then. 

There are several limitations in the present study. First, this is a retrospective case series with a relatively small sample size of 19 Korean patients. Regarding its research design, however, SFs firstly confirmed surgically were retrospectively analyzed in terms of previous history, clinical features, and surgical outcomes. Considering the rarity of SFs around the ankle joint known in the literature, we believe that such a backward approach toward limited number of SF cases detected surgically was inevitable in this study. As a result, succeeding randomized prospective comparative studies among various population would be needed to help verify the validity of this work [20,30]. Second, it is uncertain whether conservatively treated SFs related to the lateral ankle sprain or instability would eventually end up with successful clinical outcomes. All patients in this study, in fact, complained of chronic, repeated swelling around the ankle, which theoretically makes surgical closure of the SF as a preferred treatment. Still, comparative studies would verify the superiority between conservative and surgical treatment. Lastly, postoperative outcome was satisfactory with an average FAOS of 87.65.

**Figure 6:**
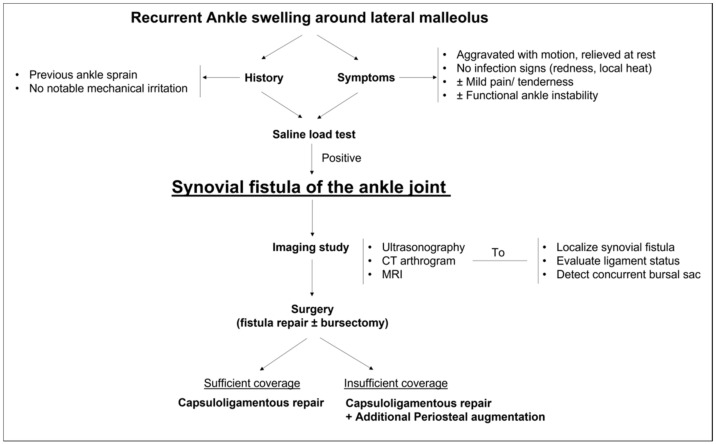
Proposal of diagnosis and treatment algorithm for synovial fistula of the ankle joint associated lateral ankle sprains or instability.

Despite these limitations, we believe that this is a valuable study to clearly highlight the fact that SF of the ankle joint can be developed after the lateral ankle sprain and should not be misdiagnosed as adventitious malleolar bursitis. From this study of our experience, we suggest a diagnostic and therapeutic algorithm for SF after the lateral ankle sprain or instability in Figure 6.

## 5. Conclusions

SF of the ankle joint needs to be taken into consideration as a possible complication in patients with a previous ankle sprain history and recurrent joint swelling. The saline load test would be useful for its diagnosis, and treatment should be focused on the complete closure of the capsular opening along with restoration of its surrounding pathologic conditions.

## Figures and Tables

**Table 1:** Descriptive data of 19 patients (yr, years; mo, months; M, male; F, female; ATFL, anterior talofibular ligament; CFL, calcaneofibular ligament; LM, lateral malleolar area; PM, premalleolar area; SF, synovial fistula; C, capsuloligamentous repair; P, periosteal augmentation; FAOS, foot and ankle outcome score).

No.	Age, yr	Sex	Time to Visitafter Trauma, mo	Saline Load Test	MRI			Bursitis (Location/SF Communication)	Surgical Procedure	FAOS	Recurrence
					+/−	ATFL	CFL				
1	59	F	3	Positive	+	Complete tear	Intact	+ (LM/−)	P	80.13	−
2	42	F	24	Positive	+	Partial tear	Partial tear	− (−/−)	C	86.75	−
3	70	M	3	Positive	−			+ (LM/−)	P	78.53	−
4	77	M	3	Positive	−			+ (PM/+)	C	77.40	−
5	75	M	3	Positive	+	Complete tear	Partial tear	+ (LM/+)	C	83.47	−
6	39	F	3	Positive	+	Partial tear	Intact	+ (PM/+)	C	92.30	−
7	62	F	4	Positive	+	Partial tear	Intact	+ (PM/+)	P	90.65	−
8	59	M	8	Positive	−			− (−/−)	C	96.74	−
9	60	F	12	Positive	+	Partial tear	Partial tear	− (−/−)	C	94.88	−
10	82	F	3	Positive	−			− (−/−)	P	98.15	−
11	74	M	6	Positive	−			+ (PM/+)	C	80.80	−
12	78	F	24	Positive	+	Complete tear	Intact	+ (PM/+)	P	77.40	−
13	52	F	3	Positive	+	Complete tear	Partial tear	− (−/−)	P	94.88	−
14	42	M	24	Positive	+	Partial tear	Intact	+ (PM/+)	Initial: C Revision: P	93.36	+
15	67	F	24	Positive	+	Partial tear	Intact	+ (PM/+)	C	92.37	−
16	40	F	84	Positive	+	Complete tear	Partial tear	− (−/−)	P	86.77	−
17	55	F	6	Positive	+	Partial tear	Partial tear	− (−/−)	C	82.47	−
18	34	M	6	Positive	-			+ (PM/-)	C	90.07	−
19	32	M	3	Positive	+	Complete tear	Partial tear	− (−/−)	C	88.38	−
Mean	57.8		12.9							87.65	

## Data Availability

The datasets used and/or analyzed during the current study are available from the corresponding author on reasonable request.
